# Integral movement therapy versus local movement therapy approach in patients with idiopathic chronic low-back pain: study protocol for a randomized controlled trial

**DOI:** 10.1186/s13063-018-3128-z

**Published:** 2019-01-21

**Authors:** Suzana Pustivšek, Nejc Šarabon

**Affiliations:** 1 Zdravstveni dom Kranj, Gosposvetska 9, 4000 Kranj, Slovenia; 2 UP Fakulteta za vede o zdravju, Polje 42, 6310 Izola, Slovenia

**Keywords:** Chronic low-back pain, Integral movement therapy, Local movement therapy, Supervised training

## Abstract

**Background:**

Chronic low-back pain (CLBP) is one of the most common reasons for seeking medical care and it imposes a significant burden on individuals and society at large. Systematic reviews evaluating the effectiveness of supervised exercise therapies commonly conclude that, to date, there is no evidence to support the superiority of one form of exercise over another. Randomized controlled trials (RCT) to date included mostly trunk strengthening exercises (e.g. bird dog, plank) and there is no evidence about supervised, individually graded integral movement therapy program for patients with CLBP.

**Methods:**

The research design is a RCT with parallel-group design including two intervention groups: integral movement therapy and conventional local movement therapy. Participants in each group will receive 20 supervised sessions in a 10-week period, twice per week, for approximately 1 h per session. Outcome assessments will occur at baseline and immediately after intervention, follow-up will take place at six months and 12 months after the intervention. Prespecified analyses will evaluate the main effects of the treatment.

**Discussion:**

This trial will use a novel, previously unexplored integral approach to CLBP through exercises. In contrast to commonly used exercise programs, the integral program does not include specific local strength exercises for hip and trunk flexors and extensors. However, learning dynamic trunk muscle control in various body positions with added limb movements could be beneficial because of the parallels to everyday work. The study will contribute to clinical practice by providing evidence to guide professionals when deciding for the proper and efficient treatment of patients with CLBP.

**Trial registration:**

ClinicalTrials.gov, NCT03623802. Registered on 9th August 2018.

**Electronic supplementary material:**

The online version of this article (10.1186/s13063-018-3128-z) contains supplementary material, which is available to authorized users.

## Background

Chronic low-back pain (CLBP) is one of the most common reasons for seeking medical care and it imposes a significant burden on individuals and society at large. The lifetime prevalence of low-back pain (LBP) is up to 84%. After an initial episode of LBP, 44–78% of people suffer relapses of pain and 26–37% relapses of work absence [1].

Literature suggests several different methods for coping with CLBP [1]. Supervised exercise therapies are among the most commonly advocated treatments for non-specific CLBP [1]. However, findings from a systematic review concluded the most effective model of exercise therapy remains uncertain [1]. Although several supervised randomized controlled trials (RCT) focusing on different approaches for managing the CLBP using movement therapy have been published so far [2–8], there is no evidence about integral movement therapy program for patients with CLBP. Systematic reviews evaluating the effectiveness of supervised exercise therapies commonly conclude that, to date, there is no evidence to support the superiority of one form of exercise over another [9, 10]. RCTs to date include mostly trunk-strengthening exercises (e.g. bird dog, plank) [5, 7, 8, 11, 12].

People with CLBP often report an impaired ability to perform daily activities. LBP can lead to a significant level of disability and physical functioning [13]. It is common for CLBP patients to avoid activity due to the fear of further exacerbating the pain or harming the spine [14]. LBP is correlated with weak core muscles and delayed muscle activation [15]. In accordance with that, we propose an integral movement therapy program, which is focused on simulating the most common daily activities, body positions, and movements, which require coordinated contraction of local and superficial muscles. Developed with expert consensus, this intervention is based on exercise progression (i.e. from stable to unstable body positions, from single-plane to multi-plane movements, and from minimal resistance/load to maximal). The main focus with all exercises and stages is the control of core/spine position.

## Methods/ design

### Aims and research questions

The primary study aim is to evaluate the efficiency of supervised and individually graded integral movement therapy program in patients with CLBP on pain, quality of life, and functional abilities. Further, the secondary aim is to compare the difference in outcome measures to supervised, conventional local movement therapy. The following research questions are addressed:

1. Does the supervised and individually graded integral movement therapy program have an effect on reducing pain, improving the quality of life and functional abilities in patients with idiopathic CLBP?
2. Does the supervised and individually graded integral movement therapy program have a clinically more significant effect on reducing pain, improving the quality of life and functional abilities compared to the supervised, conventional local movement therapy?
3. Which program has a better effect on specific core stability strength (e.g. maximal isometric trunk flexion, extension and lateral flexion) and proprioception?

### Trial design and study setting

The research design is a superiority RCT with parallel-group design allocating patients 1:1 to either integral movement therapy or conventional local movement therapy. Intervention protocol was prepared and revised by group of physiotherapy and kinesiology experts in February 2018.

The executive researcher will be Assoc. Prof. Dr. Sc. Nejc Šarabon, grad. Physioth., grad. Phys. edu. Teacher, who will communicate with other investigators, trial participants, and journals if there will be any important protocol modifications; the executive medical doctor will be Dr. Alesander Stepanović, GP; and the responsible kinesiologist who will carry on the practical part of the intervention will be Dr. Suzana Pustivšek, BSc (PE).

**Fig. 1:**
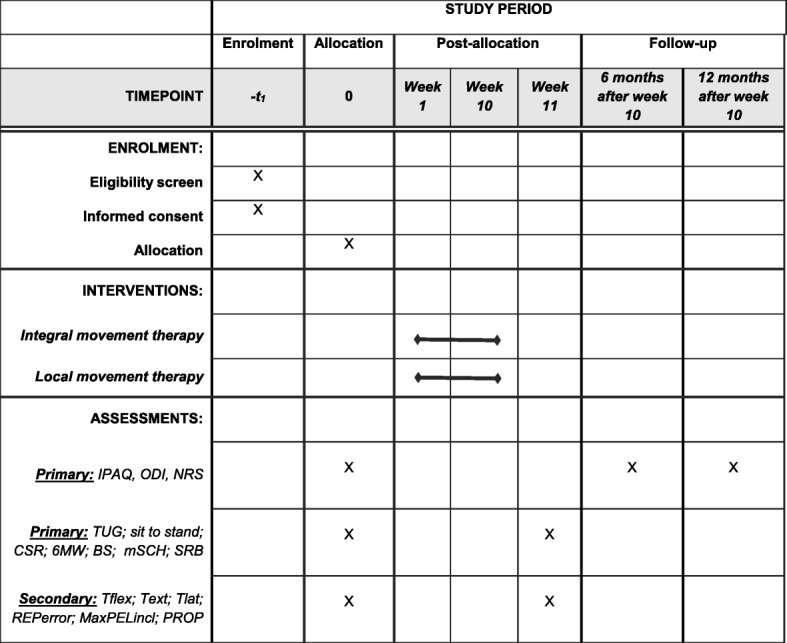
Schedule of enrollment, interventions, and assessments (Standard Protocol Items: Recommendations for Interventional Trials (SPIRIT) flow chart). IPAQ International Physical Activity Questionnaire, ODI Oswestry Disability Index questionnaire, NRS numeric rating scale, TUG timed up and go test (3 m), CSR chair seat and reach, 6MWT 6-min walk test, BS Biering-Sorensen test, mSCH Modified Schober flexibility test, SRB Sharpened Romberg balance test, Tflex trunk flexion strength, Text trunk extension strength, Tlat trunk lateral flexion strength, REPerror trunk reposition error test, MaxPELincl maximal pelvic inclination at full trunk flexion

We intend to allocate 80 adults, aged 30–60 years, into two groups. Repeated measurements will be performed at baseline and after therapy. Follow-up measures will take place six months and one year after the last therapy and include an International Physical Activity Questionnaire (IPAQ), qualitative and quantitative assessment of pain with the Oswestry Disability Index (ODI) questionnaire and the Numerical rating scale (NRS), 0–10 (see the Standards Protocol Items: Recommendations for Interventional Trials (SPIRIT) flow chart; Fig. 1). All steps and tools of the study protocol have been described adhering to the SPIRIT Check list (Additional file 1).

This study was approved by the National Medical Ethic Committee. The registration number is 0120–93/2018/6 (Additional file 2), registered on 19 March 2018, https://clinicaltrials.gov/ct2/show/NCT03623802.

The trial will be conducted in the primary healthcare unit in Kranj, the third biggest primary health center in Slovenia, under the Department of Kinesiology with cooperation from the University of Primorska, Faculty of Health Sciences.

### Sample size and sampling procedure

The sample size for this study is estimated based on clinically important improvements in patient measures through an anchor-based approach which was set at ≥ 30% [16]. In this regard, statistical power is considered to be 1-β (80%). Thus, the inclusion of at least 40 patients per group is planned [17]. Participants are allocated in a 1:1 ration to either of the two groups, local or integral, by a sealed envelope randomization system. Once the patient gives consent to enter a trial, an independent person will perform the randomization by picking one envelope out of 80, 40 of each option, and assigning the patient to the group written in the envelope.

Patients are blinded to treatment allocation, although they are informed about receiving exercise therapy without revealing the content of the treatment. Kinesiologists and physiotherapists who perform therapies are not blinded to treatment allocation, but they do not take part in the outcome measurements.

## Participants

### Recruitment

Potential participants will be identified and recruited to the trial by general practitioners who work in the healthcare center in Kranj. Patients who seek medical help due to idiopathic CLBP which persists for at least 12 weeks [1] or had at least two acute episodes of LBP in the last 12 months and fulfill the inclusion and exclusion criteria will be referred to a responsible researcher. A researcher will approach the potential participant and inform them of all aspects of the study and provide a written information sheet, which will state that participation is voluntary and that they are free to withdraw at any time without affecting subsequent medical care (Additional file 3). Personal results and treatment summary will be available at the end of the trial to each participant on individual consultation.

There are no expected contraindications during the treatment protocol, except increased LBP, which can arise in some individuals. If so, according to a consultation with the medical doctor and executive researcher, the load and intensity of exercises will be decreased. If the pain exacerbates, the patient will discontinue the program.

### Eligibility

#### Inclusion criteria

1. Idiopathic CLBP which persists for at least 12 weeks or two acute LBP episodes in the last 12 months.
2. Patients aged 30–60 years.
3. Capable of at least low physical activity to be able to complete the movement therapy program.

#### Exclusion criteria

1. Severe spinal stenosis, spondylolisthesis, fibromyalgia.
2. Lumbar spine surgery.
3. Vascular disease.
4. Neurological deficits because of nerve root or spinal cord compression.
5. Ongoing treatment for LBP.
6. Pregnancy.
7. Co-morbid health conditions that could prevent active participation in exercise.

## Interventions

Participants in each group will receive 20 supervised sessions over 10 weeks, twice per week, for approximately 1 h per session. Sessions will be carried out in small groups of up to five participants and will be supervised by an experienced kinesiologist or physiotherapist.

Both therapy programs start with a general warm-up on the elliptic trainer machine for 5 min, followed by a specific warm-up for the next 5 min including hip flexor, hip extensor, and back extensor stretching. Each stretch is repeated once and held for 30 s. Part of the warm-up routine is also learning the squat technique, pelvic-neutral position, and posture corrections. The cool-down routine includes the same stretches as the warm-up, with each stretch repeated twice (Additional file 4, part 1).

Each week, participants will receive a verbal quote of the week – known as back school. Quotes will be focused on posture, core activation and back position during lifting and carrying the loads, self-management of back pain, standing up and sitting down on the floor, pushing and pulling objects, and putting shoes on and taking them off.

Each participant will be asked to keep his/her own exercise diary in order to follow the exercise intensity: body position; color of elastic; number of sets; and repetitions in each set. Participants can move to the next level of exercise when the required number of repetitions and sets are performed without any compensatory movements of the body and with complete core stability.

### Integral movement therapy

Load and intensity of exercises will be increased according to participants’ abilities. Modifications of exercises are made by different body positions with decreasing the stability of the body position or increasing the elastic resistance. When a participant is able to perform a certain number of repetitions and sets of the required exercise without any compensatory movements, he/she can proceed to the next level of exercise. There will be 1–2-min breaks between each exercise and 20-s breaks between sets of the same exercise. In the set break, participants will perform easy trunk motions (e.g. hip circling, lateral flexion) to increase hydration of the intervertebral discs.

Protocol consists of four basic exercises, which are progressed through sessions (Additional file 4, part 2):

1. Proprioception – sitting on an unstable surface – Swiss ball, with additional tasks with legs and arms.
2. Strength – pushing task in different body positions:
  - unstable position – from single-plane to multi-plane arm movements;
  - stable position – high number of repetitions – single-plane movement.
3. Strength – pulling task in different body positions:
  - unstable position – from single plane to multi-plane arm movements;
  - stable position – high number of repetitions – single-plane movement.
4. Lifting and carrying loads:
  - stoop lifting;
  - squat lifting;
  - half kneeling lifting.

### Local movement therapy

Loads and intensity of exercises will be increased according to participants’ abilities. Modifications of exercises are made through different body positions or increasing the load. When a participant is able to perform a certain number of repetitions and sets of a required exercise, without any compensatory movements, he/she can proceed to the next level of exercise. Breaks between exercises and sets are the same as in the integral movement therapy protocol.

Protocol consists of four basic exercises, which are progressed through sessions (Additional file 4, part 3):

1. Abdomen curl.
2. Trunk extension on roman chair.
3. Hip bridge.
4. Side plank.

## Outcome measures

### Primary outcome measures

There are two aspects of primary outcome measures. The first part of the measures is based on different questionnaires: level of disability (ODI questionnaire); physical activity (IPAQ questionnaire); and pain (NRS questionnaire). All questionnaires will be conducted at baseline, immediately after the intervention, and six months and 12 months after finishing the intervention, as medium- and long-term follow-ups. The second part consists of different functional tests: timed up and go test (TUG); sit to stand test; chair seat and reach test (CSR); 6-min walk test (6MWT); Biering Sorensen test (BS); modified Schober test (mSCH); and Sharpened Romberg balance test (SRB). Those will be collected at baseline and immediately after the intervention.

The ODI questionnaire is one of the instruments for measuring disability caused by LBP. The Slovenian version of the ODI questionnaire is a reliable and valid instrument for assessing outcomes of physical therapy in patients with chronic non-specific LBP [18]. IPAQ was developed as an instrument for cross-national monitoring of physical activity and inactivity and has reasonable measurement properties for monitoring population levels of physical activity among adults in diverse settings aged 18–65 years [19]. Pain intensity is frequently measured on an 11-point pain intensity numerical rating scale, where 0 means no pain and 10 is the worst possible pain. On average, a reduction of 2 points or a reduction of approximately 30% in the NRS is considered a clinically important difference [16, 20].

TUG test, sit to stand test, CSR and 6MWT are part of the Senior Fitness Test battery [21]. Specifically, the tests measure the level of physical abilities (strength, endurance, agility) that are impaired in patients with CLBP.

Weak trunk muscles and reduced flexibility/elasticity of the back and hamstrings were found as a residual sign, in particular among those with recurrence or persistence of LBP [22]. The BS test provides reliable measures of position-holding time and can discriminate between individuals with and without non-specific LBP [23]. Furthermore, the literature states that the mSCH test showed moderate validity and excellent reliability and metrically detected changes in sample of patients with LBP [24]. SRB has been reported to have good interrater reliability and test–retest reliability [25]. Balance will be measured in three different positions of feet—parallel, semi-tandem, and tandem positions—all with closed and opened eyes.

### Secondary outcome measures

Secondary outcomes include objective measures of trunk proprioception and strength. All of them will be performed as baseline and end-of intervention measures, respectively.

Trunk repositioning error test (REPerror) will be measured for trunk flexion movement. The participant will be led to a certain angle of trunk flexion with his/her eyes covered. He/she will try to remember and repeat the targeted position of the trunk on his/her own. Flexion angle will be measured on the level of TH12 with a digital goniometer (12–075, Baseline evaluation instruments®, ZDA). The result will be expressed in degrees and will represent the difference between targeted (first angle of flexion) and actual position. Everyone will perform three sets of two repetitions. The average of repositioning errors through all repetitions will be calculated and taken for further analysis.

Furthermore, a sitting balance test will be performed. Participants will try to sit as still as possible for 35 s on the balance board, which will be placed on the force plate. Software (Fitro sway, 3.1., Fitronic s.r.o., Bratislava, Slovakia) will collect the average changes in position of center of the pressure in the anteroposterior and lateral directions and the mean velocity (mm/s) of changes of position.

The maximal strength of trunk extensors, flexors, and lateral flexors will be measured under static conditions using a dynamometer (TNC, S2P Ltd., Ljubljana, Slovenia) with an embedded force sensor (PW10AC3–200 kg, HBM, Darmstadt, Germany) [26]. The individual will stand upright with feet shoulder width apart and arms across the chest. The pelvis will be tightly fixed against the rigid support with a strap. The upper support containing the sensor will be set at shoulder height. To acquire maximal voluntary contraction force (MVC), the individual will be asked to press against the upper support as strongly as possible for 3 s. Three MVC trials (with a 20-s pause between repetitions) will be acquired for pushing forward, backward, and aside (i.e. trunk flexion, extension, and lateral flexion). The strongest among the three MVC trials (mean force on 1-s time interval) will be used for further analysis.

## Data processing and statistical analyses

All data related to the study will be stored with the highest possible level of security. Questionnaire data, functional tests results, and exercise session diaries are typed into a database for subsequent transfer into SPSS for reporting and statistical analysis. All statistical analysis will be based on an intention-to-treat basis [27]. The investigator performing the analyses is blinded to treatment allocation. Data analysis will be performed using SPSS v19 (IBM Inc., Armonk, NY, USA).

The normality of distribution will be examined for all variables and those found to have a non-normal distribution will be properly transformed before further analyses. For primary and secondary outcome scores, the mean ± SD, median and range will be given for continuous data. Comparison of pre-intervention parameters of the two groups will be determined using two sample unpaired t-test. Differences between groups in the categorical outcome variable (IPAQ) at each time point (baseline, after treatment, and six-month and 12-month follow-ups) will be analyzed using the χ^2^ test and odds ratio. Analysis of variance with repeated measures will be used to identify changes of the functional outcome variables between baseline and after therapy.

For all tests, a *P* value < 0.05 will be considered significant. The minimal clinically important differences within and between groups comparisons are defined as ≥ 30%.

There will be no interim analyses. Only the authors of the protocol and study designers, SP and NŠ, will have access to all data during the intervention.

## Discussion

The purpose of this RCT is to determine if individually graded integral movement therapy program is more effective than the widely used conventional local movement therapy program for treating patients with non-specific CLBP. This trial will use a novel, previously unexplored integral approach to CLBP through exercises. In contrast to commonly used exercise programs [5, 7, 8, 11, 12], the integral program does not include specific local strength exercises for hip and trunk flexors.

The proposed integral program is based on functional exercises with focus on trunk stability with additional single- or multi-planar limb movements. In the first few sessions, body positions are stable and limb movements are simple with minimal or without external load. The first goal is to teach participants about proper core activation in everyday body positions and movements and to build up basic proprioception, endurance, and strength. Further on, more complex tasks in less stable body positions demand more coordination and specific strength from the participant to perform it in the right way (i.e. without any compensatory movements).

It may be argued that starting a movement therapy program in an integral way is inappropriate as weak trunk muscles and delayed muscle activation are usually the main reasons for pain in patients with non-specific CLBP [15]. However, learning dynamic trunk muscle control in several body positions with added limb movements could be beneficial because of the parallels to everyday work. Literature suggests that dynamic trunk muscle control is modified during LBP remission and in patients with CLBP [28–30]. This is why learning and training core muscles in an integral way can be superior to local strengthening of the core.

As this intervention requires no expensive equipment (elastic bands and Swiss ball, optional weights), it is suitable to perform at any physiotherapy or kinesiology department. Furthermore, when patients learn to perform the exercises, they can continue performing it at home with no extra costs. The study will contribute to clinical practice by providing evidence to guide professionals when deciding the proper and efficient treatment of patients with CLBP. The results of this study will be published once the study is concluded.

## Trial status

Patient recruitment and intervention began in May 2018.

## Additional files

Additional file 1: SPIRIT 2013 Checklist: Recommended items to address in a clinical trial protocol and related documents. (DOC 121 kb)

Additional file 2: Assessment of ethical acceptability of the submitted research. (DOCX 163 kb)

Additional file 3: Consent form. (DOC 272 kb)

Additional file 4: Movement therapy protocol. Part 1: Warm-up, cool-down protocol, and back school quotes. Part 2: Integral movement therapy protocol. Part 3: Local movement therapy protocol. (DOCX 42 kb)

## Abbreviations

6MWT: 6-min walk test
BS: Biering Sorensen test
CLBP: Chronic low-back pain
CSR: Chair seat and reach
IPAQ: International physical activity questionnaire
MaxPELincl: Maximal pelvic inclination at full trunk flexion
mSCH: Modified Schober flexibility test
NRS: Numeric rating scale
ODI: Oswestry Disability Index
REPerror: Trunk reposition error test
SRB: Sharpened Romberg balance test
Text: Trunk extension strength
Tflex: Trunk flexion strength
Tlat: Trunk lateral flexion strength
TUG: Timed up and go test (3 m)
