# Supplementary material for: Integral movement therapy versus local movement therapy approach in patients with idiopathic chronic low-back pain: study protocol for a randomized controlled trial
Source: Trials. 2019 Jan 21;20:69. doi: 10.1186/s13063-018-3128-z (PMC6340171; doi:10.1186/s13063-018-3128-z)

**Assoc. Prof. Dr. Sc. Nejc Šarabon**

University of Primorska

Faculty of Health Sciences

Polje 42

6310 Izola

E-mail: [nejc.sarabon@fvz.upr.si](mailto:nejc.sarabon@fvz.upr.si)

[info@fvz.upr.si](mailto:info@fvz.upr.si)

Number: 0120-93/2018/6

Date: 19th March 2019

**Assessment of ethical acceptability of the submitted research**

Dear assoc. prof. dr. sc. Nejc Šarabon,

National Medical Ethics Committee (NMEC) has received, on 15^th^ February 2018 (dated 13^th^ February 2018), an application for ethical assessment of the research entitled ‘’Integral movement therapy versus local movement therapy approach in patients with idiopathic chronic low back pain: the effects of exercise-based interventions on trunk stabilizers strength and functional ability’’.

The first activities of this research project will begin in April 2018 and continue to the end of year 2018.

NMEC has reviewed your application during the regular session held on the 13^th^ of March 2018^1^ and concluded that the application is complete and that the proposed research is ethically acceptable. Therefore, you have received our approval to proceed with the research activities.

Yours sincerely,

dr. Božidar Voljč, MD

President of NMEC

P.S. In further correspondence regarding this research, include the approval number given above.


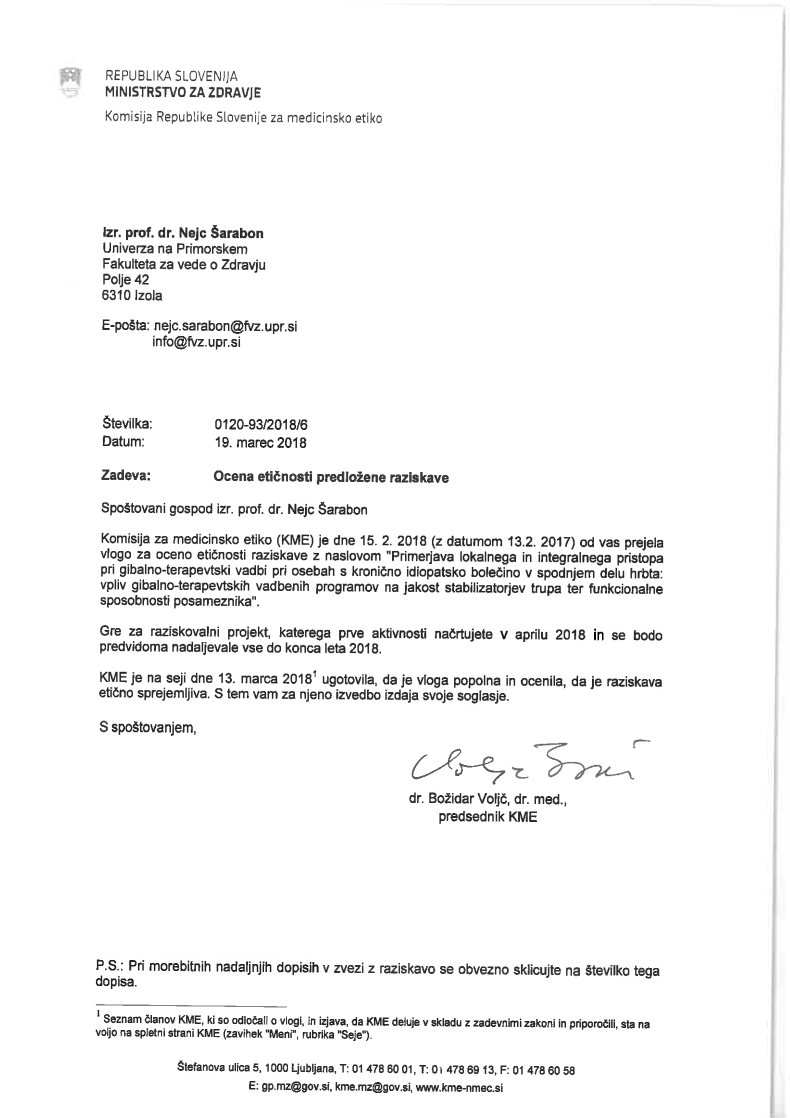

Supplement: Supplementary file 2 — Assessment of ethical acceptability of the submitted research. (DOCX 163 kb) [file 13063_2018_3128_MOESM2_ESM.docx]
