# Supplementary material for: Integral movement therapy versus local movement therapy approach in patients with idiopathic chronic low-back pain: study protocol for a randomized controlled trial
Source: Trials. 2019 Jan 21;20:69. doi: 10.1186/s13063-018-3128-z (PMC6340171; doi:10.1186/s13063-018-3128-z)
Supplement: Supplementary file 3 — Consent form. (DOC 272 kb) [file 13063_2018_3128_MOESM3_ESM.doc]

**Informed Consent form for patients o Health Centre Kranj, who are referred to exercise therapy due to chronic low back pain and are invited to participate in the research project titled:**

***INTEGRAL MOVEMENT THERAPY VERSUS LOCAL MOVEMENT THERAPY APPROACH IN PATIENTS WITH IDIOPATIC CHRONIC LOW BACK PAIN***

**This Informed Consent Form has two parts:**

- **Information Sheet (to share information about the research with you)**
- **Certificate of Consent (for signatures if you agree to take part)**

**You will be given a copy of the full Informed Consent Form**

**PART I: Information Sheet**

1. **Purpose of the research**

Chronic low back pain (CLBP) is one of the most common reasons for seeking medical care and it imposes a significant burden on individuals and society at large. The Study’s aim is to evaluate the efficiency of supervised and individually graded movement therapy programs in patients with CLBP on pain, quality of life and functional abilities.

Research is coordinated by Health centre Kranj with cooperation from the University of Primorska, Faculty of Health Sciences under the supervision of Assist. Dr. Aleksander Stepanovič, MD and Assoc. Prof. Dr. Sc. Nejc Šarabon.

1. **Voluntary Participation**

You do not have to take part in this research if you do not wish to do so. You may also stop participating in the research at any time you choose. It is your choice and all of your rights will still be respected, without any consequences.

1. **Participators tasks**

To be present on exercise sessions. To keep an exercise diary in order to follow the exercise intensity.

1. **Description of the Process**

You will receive 20 supervised sessions in 10 weeks’ time, two times per week, with approximately 1 hour per session. Sessions will be carried out in small groups, up to 5 participants and will be supervised by an experienced kinesiologist or physiotherapist.

1. **Side Effects and risk**

There are no known or expected side effects, except increased low back pain. If so, please inform the therapist about it.

1. **Benefits**

There is no material or financial benefits for participating in this research, except increasing your knowledge and experience in the field of specific exercises for chronic low back pain.

1. **Confidentiality**

The information that we collect from this research project will be kept confidential. Information about you that will be collected during the research will be put away and no-one but the researchers will be able to see it. Any information about you will have a number on it instead of your name. Only the researchers will know what your number is.

1. **Sharing the Results**

The knowledge that we get from doing this research will be shared with you through individual consultation. After finishing the trail, we will publish the results so that other interested people may learn from our research.

1. **Who to Contact**

If you have any questions you may ask them now or later, or even after the study has started. If you wish to ask questions later, you may contact any of the following: Assoc. Prof. Dr. Sc. Nejc Šarabon or PhD Suzana Pustivšek.

1. **Ethic approval**

The research protocol hes been approved by National Medical Ethics Committee. Number of approval: 0120-93/2018/6

**PART II: Certificate of Consent**

**I have read the foregoing information. I have had the opportunity to ask questions about it and any questions that I have asked have been answered to my satisfaction. I consent voluntarily to participate as a participant in this research.**

**Name and Surname of Participant__________________**

**Signature of Participant ___________________**

**Date ___________________________**

**Day/month/year**

**I confirm that the participant was given an opportunity to ask questions about the study, and all the questions asked by the participant have been answered correctly and to the best of my ability. I confirm that the individual has not been coerced into giving consent, and the consent has been given freely and voluntarily.**

**A copy of this ICF has been provided to the participant.**

**Print Name of Researcher****/person taking the consent________________________**

**Signature of Researcher /person taking the consent__________________________**

**Date ___________________________**

**Day/month/year**
