# Supplementary material for: Integral movement therapy versus local movement therapy approach in patients with idiopathic chronic low-back pain: study protocol for a randomized controlled trial
Source: Trials. 2019 Jan 21;20:69. doi: 10.1186/s13063-018-3128-z (PMC6340171; doi:10.1186/s13063-018-3128-z)
Supplement: Supplementary file 4 — Movement therapy protocol. Part 1: Warm-up, cool-down protocol, and back school quotes. Part 2: Integral movement therapy protocol. Part 3: Local movement therapy protocol. (DOCX 42 kb) [file 13063_2018_3128_MOESM4_ESM.docx]

| **Additional file 4:**  **Part 1 – Warming-up, cooling down protocol and back school quotes** | | | | | | |
| --- | --- | --- | --- | --- | --- | --- |
| **EXERCISE PROTOCOL**  **Integral movement therapy versus local movement therapy approach in patients with idiopathic chronic low back pain: study protocol for a randomized controlled trail.**  **Back school – weekly quote:**   1. Posture in standing position. 2. Relaxing positions for low back pain. 3. How to cope with sudden low back pain. 4. Load lifting – position of shoulders, back, knees, feet. 5. Carrying the loads. 6. Position of the load according to the body. 7. Putting shoes on/off. 8. Getting up from bed/couch. 9. Getting up from the floor. 10. Pushing/pulling.   **.** | | | | | | |
| **WARMING UP - general** | | | | | | |
| - Eliptic trainer – 70 % HRmax 5 min | | | | | | |
|  | | | | | | |
| - Hip flexors stretch 1x30s | | | | | | |
| - Hip extensors stretch 1x30s | | | | | | |
| - Back extensors stretch 1x30s | | | | | | |
|  | | | | | | |
| - Breathing exercises and core activation 2 min | | | | | | |
|  | | | | | | |
| **WARMING UP – specific** 2-5 min | | | | | | |
| - Squat technique teaching | | | | | | |
| - Neutral position of the spine | | | | | | |
| **Between each exercise in the main part of the session is 1-2 min break to prepare and continue on the next exercise** | | | | | | |
| **Additional file 4:**  **Part 2 – Integral movement therapy protocol** | | | | | | |
| **INTEGRAL MOVEMENT THERAPY PROTOCOL** | | | | | | |
| **NO. OF SESSION** | **1 - 6** | **Intensity variation** | **7 - 13** | **Intensity variation** | **14 - 20** | **Intensity variation** |
| **Body position** | **Sitting on a swiss ball** | | | | | |
| **Exercises** |  |  |  |  |  |  |
| **1. PROPRIOCEPTION** | 1. One leg lift |  | 1. One foott on pilates ball + other leg lift |  | 1. Both feet on pilates ball + one arm abd/add |  |
|  | 1. One leg lift + abd/add of contralateral arm | Increasing the elastic tension and colour | 1. One foot on pilates ball + other leg lift + fast abd/add of contralateral arm with 1kg weight |  | 1. One foot on pilates ball + other leg lift + fast abd/add of one arm with 1kg weight |  |
|  | - Participant continue on the next level, when he/she holds neutral position of the body 4 x 30 seconds or 2x 30 seconds left and 2x 30 seconds right side. - In between each repetition he/she performs hip circling for 20 seconds. | | | | | |
|  | | | | | | |
| **Body position** | - 1. **Push-up position** |  | **Sitting on swiss ball** |  | **Split stance** |  |
| 1. **PUSHING** | 1. Knees and elbows support | Single leg lift | 1. Double arm push | Increasing the elastic tension and colour | 1. Double arm push | Increasing the elastic tension and colour |
|  | 1. Feet and elbows support | Single arm lift | 1. Single arm push |  | 1. Single arm push |  |
|  | 1. Feet and hands support | Contralateral leg and arm lift | 1. Single arm push with contralateral leg lift |  | 1. PNF D2 flexion |  |
|  |  | Single arm lift with elastic resistance | 1. PNF D2 flexion |  |  |  |
|  | - Participant starts on the level, where he/she can perform 2 sets of 10 repetitions in required position and form without any compensatory movements. - Participant continue on the next level where he/she can perform 3 sets of 15 repetitions in required position and form without any compensatory movements. - In between each set he/she performs light trunk side flexions for 20 seconds. | | | | | |
| **Body position** | **2.2. Chair seat** |  | **Half squat** |  | **Standing** |  |
|  | 1. Double arm push | Increasing the elastic tension and colour | 1. Double arm push | Increasing the elastic tension and colour | 1. Double arm push | Increasing the elastic tension and colour |
|  | 1. Single arm push |  | 1. ingle arm push |  | 1. Single arm push |  |
|  | - Participant starts on the level, where he/she can perform 2 sets of 0 repetitions in required position and form without any compensatory movements. - Participant continue on the next level where he/she can perform 3 sets of 30 repetitions in required position and form without any compensatory movements. - 14-20 session he/she alternate between single and double arm push. - In between each repetition he/she performs hip circling for 20 seconds. | | | | | |
|  | | | | | | |

| **NO. OF SESSION** | **1 - 6** | **Intensity variation** | **7 - 13** | **Intensity variation** | **14 - 20** | **Intensity variation** |
| --- | --- | --- | --- | --- | --- | --- |
| **Body position** | **3.1. Side plank** |  | **Half kneeling** |  | **Lift from half kneeling to split stance** |  |
| **Exercises** |  |  |  |  |  |  |
| **3. 1 PULLING** | 1. Support on hip and elbow | Single arm elastic pull.  Increasing the elastic tension and colour. | 1. Double arm pull | Increasing the elastic tension and colour | 1. Double arm pull | Increasing the elastic tension and colour |
|  | 1. Support on knee and elbow |  | 1. Single arm pull (arm on kneeling side) |  | 1. Single arm pull (arm on kneeling side) |  |
|  | 1. Support on knee and extended arm |  | 1. Double arm pull , foot on pilates ball |  | 1. Double arm pull , foot on pilates ball |  |
|  | 1. Support of foot and elbow |  | 1. PNF D2 extension |  | 1. PNF D2 extension |  |
|  | - Participant starts on the level, where he/she can perform 2 sets of 10 repetitions in required position and form without any compensatory movements. - Participant continue on the next level where he/she can perform 3 sets of 15 repetitions in required position and form without any compensatory movements. - In between each repetition he/she performs knee lifting in standing position for 20 seconds. | | | | | |
| **Body position** | **3.2. Chair seat** |  | **Half squat** |  | **Standing** |  |
|  | 1. Double arm pull | Increasing the elastic tension and colour | 1. Double arm pull | Increasing the elastic tension and colour | 1. Double arm pull | Increasing the elastic tension and colour |
|  | 1. Single arm pull |  | 1. Single arm pull |  | 1. Single arm pull |  |
|  | - Participant starts on the level, where he/she can perform 2 sets of 0 repetitions in required position and form without any compensatory movements. - Participant continue on the next level where he/she can perform 3 sets of 30 repetitions in required position and form without any compensatory movements. - 14-20 session he/she alternate between single and double arm push. - In between each repetition he/she performs hip circling for 20 seconds. | | | | | |
|  | | | | | | |
| **3.2 LIFTING/ CARRYING** | 1. Double hand stoop lift | Weight on the knees height | 1. Single hand stoop lift | Lift and put down with same hand | a) Double hand stoop lift – 90° rotation – put down the weight on belt height. Repeat in reversed order | |
|  | 1. Double hand squat lift | Weight on the floor | 1. Single hand squat lift | Change hands between lift – lift with one, put down with other | b) Double hand squat lift – carry the weight for 10 m – put down on the floor | |
|  | 1. Double hand half kneeling lift |  | 1. Single hand half kneeling lift |  | c) Single hand half kneeling lift – carry the weight for 10 m over obstacles (slalom, hurdles) - put down the weight on belt height | |
|  | - Participant starts with the weight with which he/she can perform 10 repetitions in required position and form without any compensatory movements. - Participant perform 1 set of each lift (a,b,c) – up to 15 repetitions, after that the load is increased. - Maximal load is 25 kg. | | | | | |

| **Additional file 4:**  **Part 3 – Local movement therapy protocol** | | | |
| --- | --- | --- | --- |
| **Exercise** | **Intensity variation** | **No of sets and repetitions** |  |
| 1. Abdomen curl (up to 30°felxion) | Increasing the load, 2kg each time. | 2-4 x 10-20 rpt | - Participant starts on the level, where he/she can perform 2 sets of 10 repetitions in required position and form without any compensatory movements. - Participant continue on the next level where he/she can perform 4 sets of 20 repetitions in required position and form without any compensatory movements. |
| 1. Trunk extension on roman chair |  | 2-4 x 10-20 rpt |  |
| 1. Hip bridge |  |  |  |
| - 1. Heel support |  | 2-4 x 10-20 rpt |  |
| - 1. Alternating single leg extension |  |  |  |
| 3.3. Single leg hip bridge |  |  |  |
| 4. Side plank |  |  |  |
| - 1. Knee and elbow support |  | 2-4 x 10-40s | - Participant continue on the next level, when he/she holds neutral position of the body 4 x 30 seconds |
| - 1. Knee and elbow support with upper leg lifted |  |  |  |
| - 1. Extended legs – feet and elbow support |  |  |  |
| - 1. Extended legs – feet and elbow support with upper arm lifted |  |  |  |
| - 1. Extended legs – feet and elbow support with upper leg lifted |  |  |  |
